# Relationship of Test Positivity Rates with COVID-19 Epidemic Dynamics

**DOI:** 10.3390/ijerph18094655

**Published:** 2021-04-27

**Authors:** Yuki Furuse, Yura K. Ko, Kota Ninomiya, Motoi Suzuki, Hitoshi Oshitani

**Affiliations:** 1 Institute for Frontier Life and Medical Sciences, Kyoto University, Kyoto 606-8501, Japan; 2 Department of Virology, Tohoku University Graduate School of Medicine, Sendai 980-8575, Japan; yurako0603@gmail.com (Y.K.K.); oshitanih@med.tohoku.ac.jp (H.O.); 3 National Institute of Infectious Diseases, Tokyo 208-0011, Japan; ninomiya.k.aa@niph.go.jp (K.N.); mosuzuki@niid.go.jp (M.S.); 4 National Institute of Public Health, Wako 351-0197, Japan

**Keywords:** COVID-19, SARS-CoV-2, surveillance, effective reproduction number, laboratory diagnosis, epidemics, outbreaks, pandemic

## Abstract

Detection and isolation of infected people are believed to play an important role in the control of the COVID-19 pandemic. Some countries conduct large-scale screenings for testing, whereas others test mainly people with high prior probability of infection such as showing severe symptoms and/or having an epidemiological link with a known or suspected case or cluster of cases. However, what a good testing strategy is and whether the difference in testing strategy shows a meaningful, measurable impact on the COVID-19 epidemic remain unknown. Here, we showed that patterns of association between effective reproduction number (Rt) and test positivity rate can illuminate differences in testing situation among different areas, using global and local data from Japan. This association can also evaluate the adequacy of current testing systems and what information is captured in COVID-19 surveillance. The differences in testing systems alone cannot predict the results of epidemic containment efforts. Furthermore, monitoring test positivity rates and severe case proportions among the nonelderly can predict imminent case count increases. Monitoring test positivity rates in conjunction with the concurrent Rt could be useful to assess and strengthen public health management and testing systems and deepen understanding of COVID-19 epidemic dynamics.

## 1. Introduction

The pandemic of the coronavirus disease 2019 (COVID-19), caused by severe acute respiratory syndrome coronavirus 2 (SARS-CoV-2), has brought substantial morbidity and mortality in many countries. Some treatments have been shown to reduce its fatality, and several vaccines including BNT162b2 (BioNTech & Pfizer, emergency use in the USA, the EU, and so on), mRNA-1273 (Moderna, emergency use in the USA, the EU, and so on), AZD1222 (AstraZeneca, emergency use in the UK, Brazil, and so on), and Sputnik V (Gamaleya Research Institute, emergency use in Russia, Mexico, and so on) became available by the beginning of 2021 [1,2]. Notwithstanding this advance, nonpharmaceutical interventions including physical distancing, wearing a facemask, contact tracing, and testing and isolation of infected people still play a significant role in controlling the spread of the disease [3]. Many countries have implemented different kinds of public health interventions such as encouragement of remote work, school closures, bans on mass gatherings, restrictions on indoor and/or outdoor dining at restaurants/bars, and permissive to total “lockdown” stay-at-home measures, changing the level of intensity of those interventions during the course of the epidemic [4,5]. To assess the situation of infection spread and to determine the intensity of such interventions, the effective reproduction number (Rt), the average number of secondary cases resulting from contact or exposure to one infectious case, is often chosen as a key metric [6]. An Rt value >1 indicates that the number of cases is increasing, whereas an Rt of <1 suggests that the infection spread is slowing. Prediction of Rt has been extensively studied using human mobility data to forecast the number of future cases and to develop an effective strategy for public health measures [7,8]. There are also other studies that used machine learning techniques or biological data for the prediction of Rt [9,10].

Testing strategy differs among countries. Some countries conduct a population-wide or geography-wide screening to detect people with active infections, including those with no reported symptoms and those with an otherwise low expected probability of infection [11,12]. Other countries test primarily those groups with high prior probability of infection such as showing severe symptoms and/or having an epidemiological link with an identified case or cluster of cases [13]. Countries with especially limited testing capacity apply the latter, more targeted, testing strategy [14]. One theoretical study suggests that it is difficult to contain the outbreak of COVID-19 solely by implementing extensive testing for a whole population [15]; what a good testing strategy is and whether the difference in testing strategy shows a meaningful, measurable impact on the COVID-19 epidemic remain unknown [16,17,18].

Here, we analyzed an association between Rt and test positivity rate. Our results illustrate how testing has been performed for COVID-19 surveillance in Japan and across the globe. Furthermore, we found that monitoring the change in test positivity rate has the potential to predict upcoming changes in the Rt and thus provide measures to understand and monitor the dynamics of the COVID-19 epidemic.

## 2. Materials and Methods

### 2.1. Osaka Data

All confirmed COVID-19 cases in Osaka prefecture in Japan were reported to public health centers, and epidemiological and clinical information was collected from each documented case. The anonymized data of Osaka cases reported between 20 January 2020 and 15 November 2020 were provided by the Osaka prefectural government and analyzed in the present study. The raw data are available from the Osaka prefectural government upon reasonable request. We also acquired data of the numbers of total tests and positive test samples during the study period. A severe case was defined as a patient who required tracheal intubation, admission to an intensive care unit, the administration of extracorporeal membrane oxygenation, or those who died.

### 2.2. Japan Data

Data on the daily number of new confirmed cases and declared test positivity rate in each prefecture in Japan were retrieved from the website of the Ministry of Health, Labour and Welfare [19]. Japan is divided into 47 prefectures, which form the country’s first level of jurisdiction and administrative division. The population and cumulative number of COVID-19 cases in each prefecture of Japan are listed in Table S1, and 10 prefectures with the highest number of cases were selected for further analysis. Test positivity rate data in Japan were only available for the period from 9 August 2020 to 17 January 2021 and were used for analysis.

### 2.3. World Data

Daily and cumulative numbers of cases, deaths, and tests were retrieved from the Our World in Data website [20], accessed on 20 February 2021. Data for the period between 1 August 2020 and 17 January 2021 in countries that belong to the G20 were used for analysis, excluding Brazil and China because data of test positivity rate were unavailable. Because surveillance systems, including testing and reporting systems, and activities might not have been established well at the beginning of the epidemic in some countries, we analyzed global data for the period after 1 August 2020. Although vaccination started in some countries, most populations had not been vaccinated in the study period. Therefore, the vaccine effect was considered negligible in the present study.

### 2.4. Statistical Analysis

The number of reported cases, proportion of severe cases, and rate of test positivity were recomputed to be 7-day moving averages. Rt was calculated using EpiEstim with a parameter of a serial interval set to 5.19 days [21,22]. For the calculation of Rt, the earliest dates of either illness onset or infection reporting were used for Osaka data, and reporting dates were used for all other data. Spearman’s rank correlation coefficient between Rt and rate of test positivity as well as that between Rt and proportion of severe cases were calculated. Day-by-day rates of test positivity and proportions of severe cases were shifted ±20 days (by 5 days) in the analysis for the calculation of the correlation coefficient to account for time lags associated with preceding and following changes in Rt values. Data analysis and visualization were performed using R software (R Foundation for Statistical Computing, Vienna, Austria) with dplyr and ggplot2 packages [23,24].

## 3. Results and Discussion

**Figure 1:**
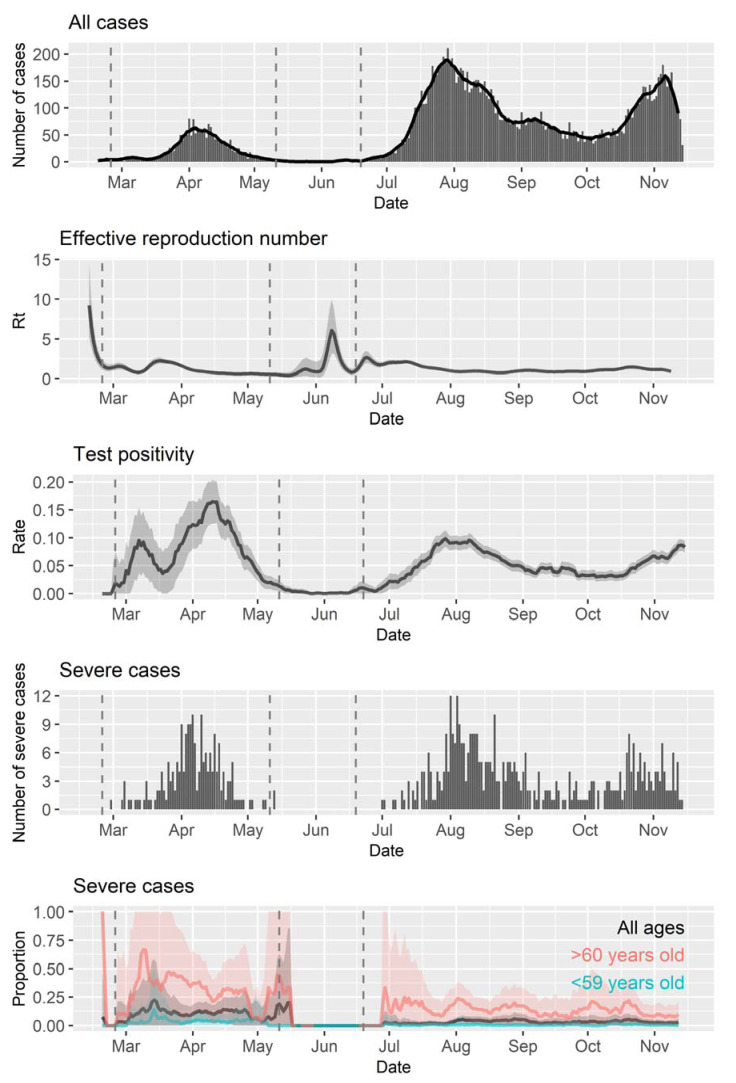
Timeline of epidemiological parameters in Osaka, Japan, from 20 January 2020 to 15 November 2020. Seven-day moving average is shown as a curved line for the number of cases. Shaded areas indicate 95% confidence intervals for Rt, rate of test positivity, and proportion of severe cases.

In Osaka, Japan, during the study period, 270,830 COVID-19 tests were conducted, and 14,864 laboratory-confirmed cases were reported, including 631 severe cases (Figure 1). The proportions of severe cases were 1.0% (116/11,655) in people ≤59 years of age (referred to as the nonelderly hereinafter) and 16.0% (515/3209) for the elderly (≥60 years of age). The number of cases was quite low from the end of May to the beginning of June, and the test positivity rate and the severe case proportion differed substantially before and after that period. Thus, to adjust and to explore further, we divided the Osaka data into two phases (Figure 1, Periods A and B) for further analysis. The strengthening of testing capacity and establishment of earlier COVID-19 treatment protocols with remdesivir and dexamethasone [25] might explain some differences observed between the periods.

**Figure 2:**
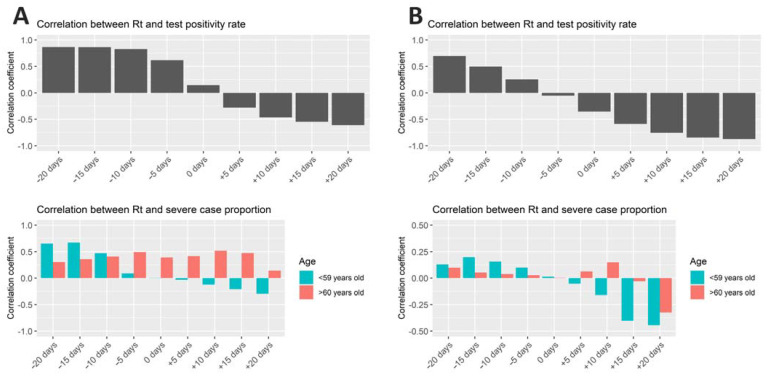
Correlation between effective reproduction number and test positivity rate/severe case proportion in Osaka. Spearman’s rank correlation coefficients between Rt and rate of test positivity (top panel) and those between Rt and proportion of severe cases (bottom panel) are shown for the Periods (**A**,**B**). Day-by-day rates of test positivity and proportions of severe cases were shifted ±20 days (by 5 days) for the calculation of correlation coefficient to see the time-lagged preceding and following associations with Rt.

In Figure 2, Spearman’s rank correlation coefficients between Rt and rate of test positivity (top panel) as well as those between Rt and proportion of severe cases (bottom panel) are shown for Periods A and B. Day-by-day rates of test positivity and proportions of severe cases were shifted ±20 days (by 5 days) for the calculation of correlation coefficient for the time-lagged preceding and following associations with Rt.

During Period A, Rt and test positivity rate showed positive correlations when the test positivity rate was considered 5–20 days toward the past (in the −5 to −20 days in the top panel of Figure 2A). A preceding change in test positivity rate suggests that an observed increase in positivity rate can predict an imminent surge in the number of cases. The positive correlation observed between Rt and test positivity rate gradually decreased and changed progressively toward the negative when the test positivity rate was shifted 5–20 days forward (in the +5 to +20 days in Figure 2A). This phenomenon can be interpreted as that more people were tested after the rise of case numbers possibly to control the disease spread by aggressive testing and isolation. The same trend was observed in Period B (Figure 2B, top panel). The proportion of severe cases in the nonelderly has a similar correlational trend with Rt, preceding positive correlation and following negative correlation (Figure 2, bottom panel), as that observed for the test positivity rate. However, that is not the case for severe case proportion in the elderly.

**Figure 3:**
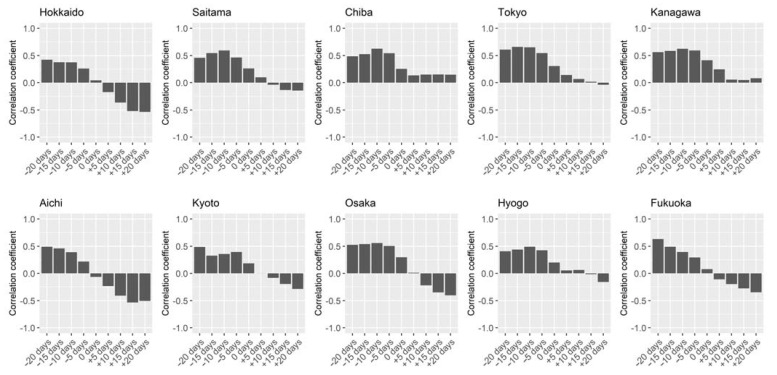
Correlation between effective reproduction number and test positivity rate in 10 prefectures in Japan. Spearman’s rank correlation coefficients between Rt and rate of test positivity are shown for 10 prefectures in Japan from 9 August 2020 to 17 January 2021. Day-by-day rates of test positivity were shifted ±20 days (by 5 days) for the calculation of correlation coefficient to see the time-lagged preceding and following associations with Rt.

Next, we tested if the association between Rt and test positivity rate existed in places outside Osaka, Japan. Data for nine other prefectures in Japan also showed similar trends across the country (Figure 3). Preceding changes of test positivity rate were present in all prefectures investigated, and following counter-changes (i.e., negative correlation) of test positivity rates compared with Rt occurred in 7 of 10 prefectures except Chiba, Tokyo, and Kanagawa. Japan’s national guidelines provide for a nationwide surveillance system [26,27]. Therefore, all prefectures would be expected to implement nearly identical public health programs with similar measures and testing strategies. As a result, the association between Rt and test positivity rate has similar patterns even in different areas in the country.

**Figure 4:**
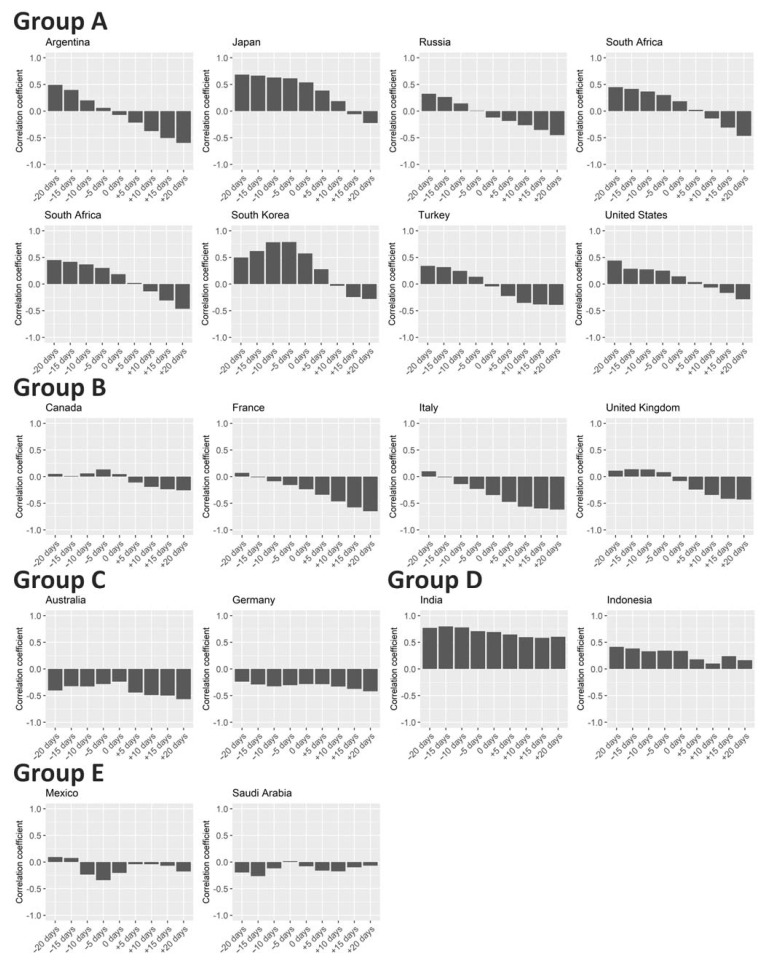
Correlation between effective reproduction number and test positivity rate in 17 countries. Spearman’s rank correlation coefficients between Rt and rate of test positivity are shown for 17 countries from 1 August 2020 to 17 January 2021. Day-by-day rates of test positivity were shifted ±20 days (by 5 days) for the calculation of correlation coefficient to see the time-lagged preceding and following associations with Rt. Countries were categorized into five groups described in the main text.

Data from other countries showed various patterns in association between Rt and test positivity rate (Figure 4). The preceding change and following counter-change in test positivity rate compared with Rt were observed in Argentina, Russia, South Africa, South Korea, Turkey, and the United States, same as in Japan (Group A). In those countries, test positivity rate can be used to predict upcoming increase in Rt. Moreover, testing intensity seemed to be affected with a delay by the situation of the epidemic there.

An epidemic situation-dependent change in testing intensity (i.e., following counter-effect) was captured in Canada, France, Italy, and the United Kingdom as well (Group B). However, preceding effects in test positivity rate to Rt were not present in those countries. Targeted populations such as those with high prior probability of infection and those with high risk of developing severe illness might have received more focus for testing in Group A countries compared with Group B countries. Targeted testing in Group A countries could have resulted in sensitive changes in test positivity rate that were followed by case trend movements.

Consistent negative correlations emerged between Rt and test positivity rate in Australia and Germany (Group C). The results for these two countries suggest that test positivity rates decreased before the increase in the case numbers and test positivity rates increased before the decrease in the case numbers. That could happen in situations where the detection of a cluster of cases triggers massive testing immediately prior to the detection of the wide spread of the infections. In contrast, there were consistent positive correlations between Rt and test positivity rate in India and Indonesia (Group D). A high positivity rate observed when the Rt is high suggests that testing was insufficient to catch up with the surge of cases. No clear correlations were detected for Mexico and Saudi Arabia (Group E).

**Figure 5:**
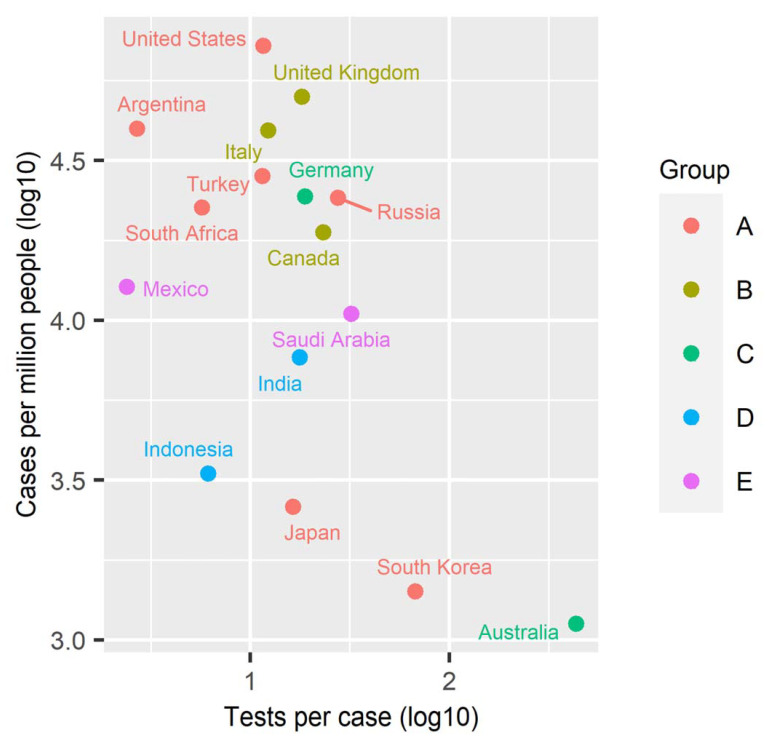
Association with the numbers of cases and tests. The total number of tests per case and the total number of cases per million people in each country reported by 17 January 2021 were plotted with colors according to the groups described in Figure 4. France was excluded from the panel because data of the total number of tests were not available.

Finally, we checked relationships between the classification based on the association between Rt and test positivity rate (Groups A–E) and the numbers of cases and tests, in each country (Figure 5). There was a moderate but nonsignificant negative correlation between the number of tests per case and the number of cases per capita (Spearman’s rho = −0.40; p-value = 0.13). However, there was no clear association between the group classification and the number of cases or tests. The results indicate that any differences in association between Rt and test positivity rate were not simply caused by absolute magnitude of testing intensity. Moreover, difference in the association between Rt and test positivity rate had no obvious impact on the size of the COVID-19 epidemic.

Some countries and regions in Western Pacific Region such as New Zealand, Taiwan, and Thailand, in addition to Australia, which was investigated in the previous analysis shown in Figure 5, have conducted testing with great intensity and have kept the COVID-19 case numbers quite low (Table S2) [28,29]. Other countries in the area, such as Japan, Malaysia, Philippines, Singapore, and South Korea, where testing intensity was not as high as that of the aforementioned countries, also have relatively small numbers of cases compared to countries in North America and Europe (Figure 5 and Table S2). Socio-economic environments may have affected testing systems, as we saw two countries in the South-East Asia Region, India and Indonesia, were classified in the same group (Figure 4). However, it is also interesting that low- and middle-income countries have not always suffered from a high disease burden of COVID-19 (Figure 4 and Figure 5, Table S2) [30,31,32]. A recent study from Taiwan suggests that both case-based and population-based interventions including testing are required for COVID-19 containment [33].

## 4. Conclusions

To the best of our knowledge, this is the first study to investigate the association between test positivity rate and epidemic trend. We showed that patterns of association between Rt and test positivity rate can illuminate differences in testing situation among different areas. This association can also evaluate the adequacy of current testing systems and what information is captured in COVID-19 surveillance. We found that the differences in testing systems alone cannot predict the results of epidemic containment efforts.

Furthermore, we found that monitoring test positivity rate can be a tool to predict imminent case count increases (in Group A countries). Data from Osaka suggest the proportion of severe disease cases in the nonelderly can serve as a surrogate measure of the test positivity rate for that purpose. The results suggest two possibilities: (1) the high proportion of severe cases in the nonelderly reflects a high positivity rate possibly caused by many mild and asymptomatic cases being missed in the surveillance and (2) the undiagnosed nonelderly are the driving force of the disease spread, although the present study cannot confirm these possibilities or elucidate any causality insights about the community transmission of COVID-19. In contrast, the infections in the elderly seem less likely to be missed and/or they play a smaller role in the disease spread because the proportion of severe disease cases in the elderly did not show the particular pattern in association with Rt. Our previous study also revealed the importance of asymptomatic/presymptomatic infected nonelderly in the generation of clusters of cases in communities [34].

Because Rt is calculated retrospectively using data of the number of infected people reported, the prediction of future Rt using test positivity rate, or severe case proportion, would help us more closely consider the adequacy and applicability of public health measures. Limitations of the present study include that we did not consider the impact or other effects of public health interventions, vaccination, people’s mobility, seasonality, or differences of viral variants [4,35,36,37,38]. Rather, the effects of those factors were considered integrated into Rt reflecting the trend of case numbers.

Current surveillance and testing systems cannot detect all infected cases in real time [39,40]. In this study, we present a method to assess how testing has been performed for COVID-19 surveillance. Future studies should attempt to predict the trajectory of the COVID-19 epidemic and identify target populations to be strategically tested, using data on test positivity rate. Monitoring test positivity rates could be useful to evaluate and strengthen public health management and testing systems and deepen understanding of COVID-19 epidemic dynamics.

## Data Availability

The data presented in this study are available on request from the corresponding author. The Osaka data are not publicly available due to an agreement with the Osaka prefectural government.

## Supplementary Materials

The following are available online at https://www.mdpi.com/article/10.3390/ijerph18094655/s1, Table S1: Cumulative number of cases and population in 47 prefectures in Japan, Table S2: Numbers of tests and cases in countries and region in Western Pacific Region.
