# Supplementary material for: Relationship of Test Positivity Rates with COVID-19 Epidemic Dynamics
Source: Int J Environ Res Public Health. 2021 Apr 27;18(9):4655. doi: 10.3390/ijerph18094655 (PMC8125747; doi:10.3390/ijerph18094655)
Supplement: Supplementary file 1 [file ijerph-18-04655-s001.zip › ijerph-1193917-supplementary.pdf]

Supplementary tables

Prefecture	Cumulative cases[*1]	Cumulative deaths[*1]	Population[*2] (million)	Cases per million people	Deaths per million people
Tokyo	85541	725	13.921	6144.7	52.1
Osaka	38160	742	8.809	4331.9	84.2
Kanagawa	34209	353	9.198	3719.2	38.4
Saitama	21599	269	7.35	2938.6	36.6
Aichi	21312	317	7.552	2822.0	42.0
Chiba	18383	169	6.259	2937.1	27.0
Hokkaido	15969	538	5.25	3041.7	102.5
Hyogo	13956	306	5.466	2553.2	56.0
Fukuoka	13446	136	5.104	2634.4	26.6
Kyoto	6916	78	2.583	2677.5	30.2
Okinawa	6511	86	1.453	4481.1	59.2
Hiroshima	4409	70	2.804	1572.4	25.0
Shizuoka	3933	63	3.644	1079.3	17.3
Ibaraki	3888	44	2.86	1359.4	15.4
Gifu	3542	55	1.987	1782.6	27.7
Gunma	3325	60	1.942	1712.2	30.9
Tochigi	3213	18	1.934	1661.3	9.3
Kumamoto	2987	38	1.748	1708.8	21.7
Miyagi	2973	20	2.306	1289.2	8.7
Nara	2567	31	1.33	1930.1	23.3
Okayama	2102	16	1.89	1112.2	8.5
Nagano	2016	21	2.049	983.9	10.2
Mie	1800	23	1.781	1010.7	12.9
Shiga	1766	19	1.414	1248.9	13.4
Miyazaki	1567	11	1.073	1460.4	10.3
Fukushima	1469	33	1.846	795.8	17.9
Kagoshima	1386	15	1.602	865.2	9.4
Ishikawa	1339	54	1.138	1176.6	47.5
Nagasaki	1266	14	1.327	954.0	10.6
Oita	933	11	1.135	822.0	9.7
Wakayama	885	11	0.925	956.8	11.9
Yamaguchi	856	5	1.358	630.3	3.7
Yamanashi	854	11	0.811	1053.0	13.6
Toyama	813	27	1.044	778.7	25.9
Ehime	812	14	1.339	606.4	10.5
Kochi	779	13	0.698	1116.0	18.6
Niigata	775	5	2.223	348.6	2.2
Saga	766	4	0.815	939.9	4.9
Aomori	644	8	1.246	516.9	6.4
Kagawa	537	5	0.956	561.7	5.2
Iwate	471	25	1.227	383.9	20.4
Fukui	451	12	0.768	587.2	15.6
Yamagata	443	13	1.078	410.9	12.1
Tokushima	297	9	0.728	408.0	12.4
Shimane	237	0	0.674	351.6	0.0
Akita	216	1	0.966	223.6	1.0
Tottori	178	2	0.556	320.1	3.6

Table S1. Cumulative number of cases and population in 47 prefectures in Japan.

*1 As of 17 January 2021

*2 As of 1 October 2019. Data were obtained from the website of Statistics Bureau, Ministry of Internal Affairs and Communications, Japan (https://www.stat.go.jp/data/jinsui/2019np/index.html)

Country or region	Cases per million people	Tests per case
Australia	1126.3	431.9
Japan	2614.8	16.4
Malaysia	4895.1	25.2
New Zealand	469.1	647.2
Philippines	4568.1	13.8
Singapore	10104.2	102.5
South Korea	1418.6	67.4
Taiwan	35.9	160.8
Thailand	178.0	165.9

Table S2. Numbers of tests and cases in countries and region in Western Pacific Region.

Data as of 17 January 2021
